# Antioxidant Regulation of Cell Reprogramming

**DOI:** 10.3390/antiox8080323

**Published:** 2019-08-20

**Authors:** Yuichiro J. Suzuki, Nataliia V. Shults

**Affiliations:** Department of Pharmacology and Physiology, Georgetown University Medical Center, Washington, DC 20007, USA

**Keywords:** antioxidants, pluripotency, reactive oxygen species, reprogramming, stem cells

## Abstract

Discovery of induced pluripotent stem cells (iPSCs) has revolutionized regeneration biology, providing further mechanistic insights and possible therapeutic applications. The original discovery by Yamanaka and co-workers showed that the expression of four transcription factors in fibroblasts resulted in the generation of iPSCs that can be differentiated into various cell types. This technology should be particularly useful for restoring cells with limited proliferative capacities such as adult heart muscle cells and neurons, in order to treat diseases affecting these cell types. More recently, iPSCs-mediated cell reprogramming has advanced to new technologies including direct reprogramming and pharmacological reprogramming. Direct reprogramming allows for the conversion of fibroblasts into cardiomyocytes, neurons or other cells by expressing multiple cell type-specific transcription factors without going through the production of iPSCs. Both iPSC-mediated reprogramming as well as direct reprogramming can also be promoted by a combination of small molecules, opening up a possibility for pharmacological therapies to induce cell reprogramming. However, all of these processes have been shown to be affected by reactive oxygen species that reduce the efficacies of reprogramming fibroblasts into iPSCs, differentiating iPSCs into target cells, as well as direct reprogramming. Accordingly, antioxidants have been shown to support these reprogramming processes and this review article summarizes these findings. It should be noted however, that the actions of antioxidants to support cell reprogramming may be through their ROS inhibiting abilities, but could also be due to mechanisms that are independent of classical antioxidant actions.

## 1. Introduction

In 2012, Prof. Shinya Yamanaka won the Nobel Prize for his discovery that somatic cells can be converted into stem cell-like cells [1]. Specifically, ectopic expression of four stem cell transcription factors to fibroblasts resulted in cells with different characteristics being differentiated into various cell types such as neuronal cells, hepatocytes, cardiac myocytes, and hematopoietic progenitor cells [1,2,3]. These stem cell-like cells were named induced pluripotent stem cells (iPSCs) and have revolutionized regeneration biology in terms of possible therapeutic strategies as well as providing cells that may be useful for research [1]. Such concepts have advanced to the next generation of technologies in regenerative medicine, including direct cell reprogramming where for example, combinations of cardiac or neuronal transcription factors directly convert fibroblasts into cardiomyocytes [4,5,6] or neurons [7,8,9] respectively. Further, in pharmacological reprogramming, instead of ectopically expressing transcription factors, combinations of small molecules can promote similar cell reprogramming events [10,11,12,13,14,15].

Accumulating evidence suggests that oxidative stress and reactive oxygen species (ROS) negatively influence the development of iPSCs and the differentiation of iPSCs into target cells, as well as the direct reprogramming events [16]. These results suggest that antioxidants may be therapeutically useful in supporting cell reprogramming-based therapeutic strategies, opening up a new use of antioxidants in biomedicine. This review article compiles experimental evidence for the effects of antioxidants in cell reprogramming therapies for the purpose of helping design effective therapeutic strategies to use cell reprogramming technologies in combination with antioxidants.

## 2. Discovery of iPSCs

**Figure 1:**
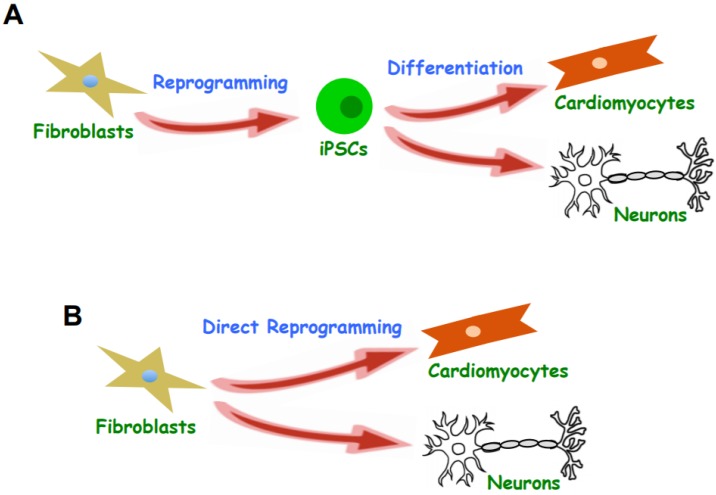
Schematics of cellular reprogramming. (**A**) Fibroblasts get converted into induced pluripotent stem cells (iPSCs) that in turn get differentiated into cardiomyocytes or neurons. (**B**) Fibroblasts directly get converted into cardiomyocytes or neurons.

**Figure 2:**
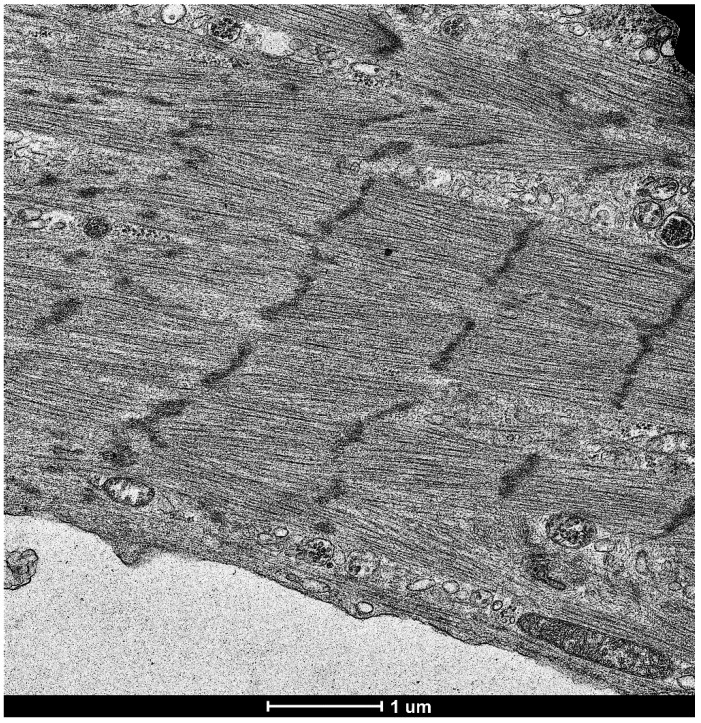
Transmission electron microscopy image of cardiomyocytes produced from fibroblast-derived iPSCs. The reprogramming technology was used to convert cultured human fibroblasts into iPSCs and then into cardiomyocytes (Cell Applications, Inc., San Diego, CA, USA). Cells were then fixed in the 2.5% glutaraldehyde/0.05 M cacodylate solution, post-fixed with 1% osmium tetroxide and embedded in EmBed812 (Electron Microscopy Sciences Hatfield, PA, USA). Ultrathin sections (70 nm) were post-stained with uranyl acetate and lead citrate and examined in the Talos F200X FEG transmission electron microscope (FEI, Hillsboro, OR, USA) [18]. The image shows the cells developed from fibroblasts having clear striations and sarcomere structures. Magnification 5500×.

Cellular reprogramming is defined as the conversion of one specific cell type to another. This technology gained considerable attention when Prof. Yamanaka discovered the means to convert fibroblasts into iPSCs [1,2,3]. In these studies, forced expression of four stem cell-related transcription factors (Oct3/4, Sox2, c-Myc and Klf4) was found to convert somatic cells into pluripotent cells that can be differentiated into various cell types including cardiomyocytes, neurons (Figure 1A) and various other cell types [1]. Our transmission electron microscopy (TEM) experiments confirmed that induced cardiomyocytes derived from iPSCs indeed exhibit organized sarcomere structures with clear striations and Z-lines (Figure 2). The iPSC technology can be useful for individualized medicine in which patients’ skin fibroblasts are converted to iPSCs that are in turn, returned to the same patients to reconstruct the myocardium, the nervous system and other tissues/organs without the limitations of the tissue rejection [1]. Since its discovery, iPSCs has been widely used for disease modeling, drug discovery and cell therapies [17].

## 3. Direct Reprogramming

More recently, the introduction of a combination of cardiac or neuronal transcription factors was found to directly convert fibroblasts into cardiomyocytes [4,5,6] or neurons [7,8,9], respectively. This reprogramming event that is not mediated by the production of iPSCs has been termed “direct reprogramming” (Figure 1B). The combination of cardiac transcription factors that promote direct reprogramming of fibroblasts into cardiomyocytes included GATA4, MEF2C, TBX5 and HAND2 [4,5,6]. A combination of *Ascl1*, *Brn2* (also called *Pou3f2*) and *Myt1l* were found to reprogram fibroblasts into neurons [7,8]. Treutlein et al. [9] used single-cell RNA-seq to dissect direct reprogramming mechanisms from fibroblasts to neurons. Direct reprogramming can also generate other cell types such as myoblasts, hematopoietic progenitor cells and pancreatic beta cells [10].

## 4. Pharmacological Reprogramming

In addition to expressing multiple transcription factors, combinations of small molecules have also been shown to elicit cell reprogramming of fibroblasts either into iPSCs or directly to target cells such as cardiomyocytes and neurons. This event is now known as “pharmacological reprogramming”. The use of small molecules to promote cell reprogramming is appealing in that it is easier to use and more cost effective pharmacological therapies may be adapted in the reprogramming technology. After screening up to 10,000 small molecules, Hou et al. [11] initially discovered that a combination of seven small-molecule compounds can convert fibroblasts into pluripotent stem cells. Subsequently, similar approaches using a combination of small molecules were found to promote direct reprogramming of fibroblasts to cardiomyocytes [12,13] or to neurons [14,15,16]. The advancement of pharmacological reprogramming into other cell types is limited.

## 5. Discovery of Effects of Vitamin C on Differentiation of Embryonic Stem Cells into Cardiomyocytes

In 2003, Takahashi et al. [17] published a study, which screened a drug library for the differentiation of embryonic stem cells into α-myosin heavy chain promoter active cardiomyocytes. Among 880 compounds tested, vitamin C (ascorbic acid) was the only compound that reproducibly induced the differentiation. The treatment of embryonic stem cells with vitamin C also induced the expression of cardiac genes such as GATA-4, α-myosin heavy chain and β-myosin heavy chain. This reprogramming ability was found to be unique to vitamin C, whereas other antioxidants such as *N*-acetylcysteine, Tiron or vitamin E did not cause the differentiation. These results reveal that vitamin C, either through its non-antioxidant actions or via its specific antioxidant mechanism, can influence cell reprogramming from embryonic stem cells into cardiomyocytes. This study has become a foundation for other studies investigating the effects of vitamin C and other antioxidants on cell reprogramming that involves iPSCs and the direct reprogramming processes.

## 6. Effects of Vitamin C on iPSC Production

Pei and co-workers [19] first demonstrated the generation of ROS during the reprogramming of fibroblasts to iPSCs. Specifically, they noted that the ROS production in cells transduced with Sox2/Klf4/Oct4, but not with Sox2/Klf4/Oct4/c-Myc. Since Sox2/Klf4/Oct4 is less efficient in producing iPSCs compared to Sox2/Klf4/Oct4/c-Myc, ROS may be inhibiting Sox2/Klf4/Oct4-mediated reprogramming. The authors further found that vitamin C increased the efficacy of Sox2/Klf4/Oct4-mediated reprogramming [18]. Interestingly, other molecules antioxidant activities including vitamin B1 (thiamine), reduced glutathione, sodium selenite, *N*-acetylcysteine, resveratrol, α-lipoic acid, vitamin E and L-carnitine did not support the reprogramming process [19]. Thus, it is unclear in their study if the antioxidant action of vitamin C is involved in this event.

Further experiments by Pei and co-workers [20] supported the concept that vitamin C activates jumonji-domain containing histone demethylases *Jhdm1a/1b* that in turn, induces demethylation of H3K36me2/3 and accelerates reprogramming. This is supported by their observation that the overexpression of *Jhdm1a/1b* enhanced reprogramming by Sox2/Klf4/Oct4. Since this study did not address the molecular mechanism of vitamin C-induced activation of *Jhdm1a/1b*, it is yet unclear if this process is related to the role of ROS in cell reprogramming. Vitamin C has also been shown to prevent the loss of Dlk1-Dio3 imprinting [21] and modulates TET1 function [22] to enhance reprogramming.

## 7. Effects of Other Antioxidants on iPSC Production

Other molecules with antioxidant properties that have been shown to enhance the reprogramming of fibroblasts into iPSCs include resveratrol [23,24], *N*-acetylcysteine [25], EUK134, ebselen, Mito-TEMPO, and NADPH oxidase inhibitors [26]. Experiments by Ji et al. [25] showed that forced expression of Oct4, Sox2, Klf4 and c-Myc in human fibroblasts resulted in increased ROS production and DNA double-strand breaks. An antioxidant *N*-acetylcysteine reduced these events, suggesting that ROS-induced DNA damage results in genomic aberrations in iPSCs.

## 8. Effects of Antioxidants on the Differentiation of iPSCs

In addition to the ability of vitamin C to promote the reprogramming of fibroblast into iPSCs, this molecule has been shown to support the differentiation of iPSCs into cardiac cells. Cao et al. [27] systematically screened 16 cytokines and chemicals and found that vitamin C was the only factor that enhanced the cardiac differentiation of iPSCs. The mechanism of vitamin C was found to involve the collagen synthesis-dependent proliferation of cardiac progenitor cells via the MEK-ERK1/2 pathway. Similarly, melatonin that possesses antioxidant properties has been reported to increase the neural differentiation of iPSCs by activating the phosphoinositide 3-kinase/Akt pathway [28].

## 9. Effects of Antioxidants on Direct Reprogramming

Similarly, direct reprogramming of fibroblasts into cardiomyocytes was found to be promoted by antioxidants. Talkhabi et al. [29] reported that vitamin C enhances the direct conversion of mouse fibroblasts into cardiomyocytes induced by Yamanaka factors plus small molecules. Selenium was found to promote microRNA-directed direct reprogramming of fibroblasts into cardiomyocytes [30]. Gascón et al. [31] showed that lipid peroxidation is increased during direct reprogramming into neurons. The induction of oxidative stress during direct neuronal reprogramming was confirmed by observing the occurrence of dimeric peroxiredoxin 2 through disulfide linkage. Their experiments also suggested that oxidative stress-induced ferroptosis is the limiting factor during neuronal reprogramming. Using the transcriptome analysis, authors revealed some oxidative stress response mechanisms to be enhanced, including the Nrf-2 pathway. Further, they showed that the conversion of reactive glia into neurons was improved by vitamin E, α-tocotrienol, or by activating antioxidant pathways by calcitriol [31].

## 10. Effects of Antioxidants on Pharmacological Reprogramming In Vivo

**Figure 3:**
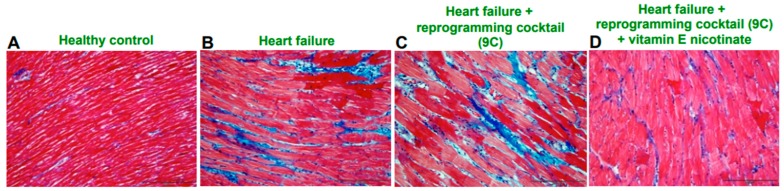
Effects of pharmacologic reprogramming drug cocktail (9C) and vitamin E nicotinate on cardiac fibrosis. Sprague-Dawley rats were subcultaneously injected with SU5416 (20 mg/kg body weight) and subjected to chronic hypoxia at 10% O_2_ for 3 weeks and then maintained in normoxia to promote pulmonary hypertension-induced right ventricular fibrosis [32]. Eight weeks after the injection of SU5416, a combination of cell reprogramming drugs (9C) as described by Cao et al. [13] was injected intraperitoneally with or without vitamin E nicotinate twice over the one-week period. Right ventricular tissues were fixed in buffered formalin, embedded in paraffin, and subjected to Masson’s trichrome staining. Blue color indicates fibrosis. (**A**) Healthy control; (**B**) Heart failure; (**C**) Heart failure + reprogramming cocktail (9C); (**D**) Heart failure + reprogramming cocktail (9C) + vitamin E nicotinate. Magnification 400×.

**Figure 4:**
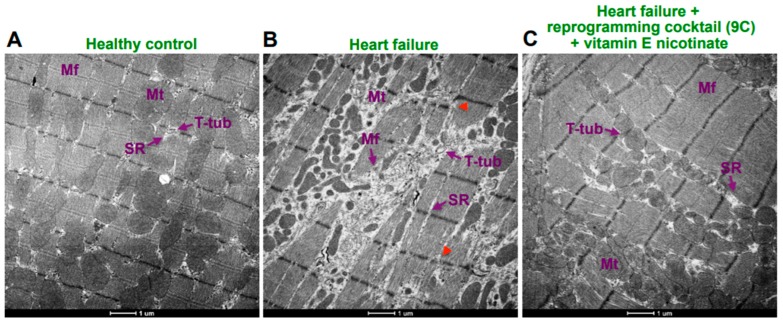
Effects of pharmacologic reprogramming drug cocktail (9C) plus vitamin E nicotinate on the cardiac muscle ultrastructure. SU5416-injected Sprague-Dawley rats were subjected to 3 weeks hypoxia and then maintained in normoxia to promote pulmonary hypertension-induced right ventricular damage [32]. Eight weeks after the injection of SU5416, a combination of cell reprogramming drugs (9C) plus vitamin E nicotinate were injected intraperitoneally twice over the one-week period. Tissues were fixed in the 2.5% glutaraldehyde/0.05 M cacodylate solution, post-fixed with 1% osmium tetroxide and embedded in EmBed812. Ultrathin sections (70 nm) were post-stained with uranyl acetate and lead citrate and examined in the Talos F200X FEG transmission electron microscope (FEI, Hillsboro, OR, USA) [18]. Transmission electron microscopy image represents (**A**) normal ultrastructure of the right ventricular cardiomyocyte of a healthy rat: Mf—myofibrils, Mt—mitochondria, T-tub—tubular system, SR—sarcoplasmic reticulum. (**B**) TEM image of the failing heart due to pulmonary hypertension shows disruptive changes to structural organization of the right ventricular cardiomyocyte, such as damage to the myofilaments (Mf), mismatches of Z-lines with their focal absence (red arrowheads), damage to mitochondria (Mt), alteration to T-tubular system (T-tub) and the sarcoplasmic reticulum (SR). (**C**) TEM image of the heart of rats with pulmonary hypertension treated with reprogramming cocktail (9C) plus vitamin E nicotinate demonstrates the restoration of ultrastructure of the right ventricular cardiomyocyte. Myofibrils (Mf), mitochondria (Mt), T-tubular system (T-tub) and sarcoplasmic reticulum (SR) have same structure with normal. Magnification 5500×.

To our knowledge, effects of antioxidants on in vivo pharmacological reprogramming have not been reported. Thus, we performed preliminary experiments to determine the effects of vitamin E nicotinate on pharmacological reprogramming in rats. The Georgetown University Animal Care and Use Committee approved all the animal experiments (ethical code: 2017-0043). Our investigation conformed to the National Institutes of Health Guide for the Care and Use of Laboratory Animals. To test the hypothesis that the pharmacological reprogramming cocktail of small molecules can repair the heart, we injected Sprague-Dawley rats with pulmonary hypertension with a cocktail of 9 small molecules (9C) that have been shown to elicit cell reprogramming converting fibroblasts into cardiomyocytes in culture [13]. Pulmonary hypertension was induced by the injection of SU5416 (an inhibitor of vascular endothelial growth factor receptor) and chronic hypoxia that promotes severe right ventricular fibrosis [32]. After pulmonary hypertension and right ventricular fibrosis were developed, the 9C cocktail including CHIR99021 (GSK3 inhibitor; 2 mg/kg body weight), A83-01 (ALK5 inhibitor; 2 mg/kg), SC1 (ERK1 and Ras GTPase inhibitor; 2 mg/kg), OAC2 (Activator of transcription of reprogramming factor Oct4; 2 mg/kg), Y27632 (ROCK inhibitor; 2 mg/kg), BIX01294 (histone methyl transferase inhibitor; 2 mg/kg), AS8351 (histone demethylase inhibitor; 2 mg/kg), SU16F (PDGF receptor-beta inhibitor; 2 mg/kg) and JNJ10198409 (PDGFR inhibitor; 2 mg/kg), which has been shown to reprogram fibroblasts into beating induced cardiomyocytes in culture [13], was injected twice over the one-week period. Animals were euthanized and the tissues were obtained one week after the first administration of the 9C cocktail. Heart tissues were fixed in formalin and embedded in paraffin for Masson’s trichrome staining to monitor fibrosis. Results show that pulmonary hypertension-induced right heart failure is associated with the development of fibrosis (blue staining in Figure 3B). The cocktail did not provide beneficial effects, rather worsened the right heart damage in animals with pulmonary hypertension (Figure 3B vs. Figure 3C). To test the effects of antioxidants, we added α-tocopheryl nicotinate (vitamin E nicotinate; 2 mg/kg body weight) to the 9C cocktail. With this treatment, cardiac fibrosis induced by pulmonary hypertension was almost completely eliminated (Figure 3B vs. Figure 3D). Transmission electron micrographs show that the right ventricular muscle ultrastructure that was distorted by pulmonary hypertension (Figure 4A vs. Figure 4B) was completely restored by ‘a cocktail of nine small molecules + vitamin E nicotinate’ (Figure 4B vs. Figure 4C). These preliminary and unpublished results endorse the notion that antioxidants support the pharmacological reprogramming strategies to repair the heart damage in vivo, and encourages future experiments to define the role of antioxidants in cell reprogramming in vivo, using pharmacological reprogramming strategies that can readily be performed in experimental settings.

While vitamin E nicotinate is expected to serve as a source of active vitamin E antioxidant [33], vitamin E nicotinate could also exert biological actions independent of serving as a source of vitamin E as described in an accompanied article of this Special Issue [34]. Thus, further work is needed to determine if the observed effects are indeed due to the elimination of ROS.

## 11. Conclusions

**Figure 5:**
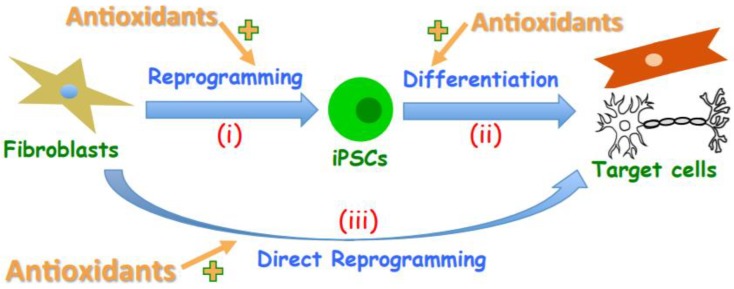
Schematics of the effects of antioxidants on cellular reprogramming. Antioxidants can improve (i) the conversion of fibroblasts into iPSCs, (ii) the differentiation of iPSCs into target cells, and (iii) the direct conversion of fibroblasts into target cells.

Cell reprogramming technologies may be promising for serving as therapeutic strategies to regenerate cells, particularly those that are not capable of readily dividing such as adult heart muscle cells and neurons. However, biological oxidation may limit the successful reprogramming processes and thus the inclusion of antioxidants may be beneficial to support the ability of reprogramming agents to produce desired functional cells. Antioxidants have been shown to improve (i) the conversion of fibroblasts into iPSCs, (ii) the differentiation of iPSCs into target cells, and (iii) the direct conversion of fibroblasts into target cells (Figure 5). Whilst some molecules that are traditionally known as antioxidants can provide benefits to reprogramming strategies, it is not yet clear if these effects are indeed due to antioxidant actions rather than activities that are independent of affecting biological oxidants. Further work that elucidates mechanisms of these antioxidant and/or non-antioxidant actions of these molecules should provide important information for developing novel therapeutic strategies to treat currently incurable medical conditions through cell reprogramming technologies.
